# Vestibular paroxysmia caused by contralateral tortuous vertebral artery: A case report

**DOI:** 10.1097/MD.0000000000027815

**Published:** 2021-11-12

**Authors:** Jin Woo Choi, Chang-Hee Kim

**Affiliations:** a Department of Radiology, Konkuk University Medical Center, Research Institute of Medical Science, Konkuk University School of Medicine, Seoul, Republic of Korea; b Department of Otorhinolaryngology-Head and Neck Surgery, Konkuk University Medical Center, Research Institute of Medical Science, Konkuk University School of Medicine, Seoul, Republic of Korea.

**Keywords:** hemifacial spasm, nystagmus, tinnitus, vertebrobasilar dolichoectasia, vestibular paroxysmia

## Abstract

**Rationale:**

Vestibular paroxysmia (VP) is characterized by spontaneous, recurrent, short, paroxysmal attacks of vertigo with or without tinnitus.

**Patient concerns:**

We report a case of paroxysmal recurrent vertigo accompanying clicking tinnitus on the left side in a 61-year-old patient. He had undergone microvascular decompression to treat the left-side hemifacial spasm 6 years prior. The patient first developed vertigo attacks about 3 years after microvascular decompression, and the attacks increased in frequency over the last 4 months. Video-nystagmography revealed a background right-beating nystagmus which was reversed every 55 seconds, to left-beating nystagmus for 17 seconds.

**Diagnosis:**

Brain magnetic resonance imaging and angiography demonstrated a compression of the cisternal segment of the left vestibulocochlear nerve between the tortuous right vertebral artery and the posterior wall of the left porus acusticus internus.

**Interventions and outcomes:**

Under the diagnosis of VP, 300 mg oxcarbazepine was administered daily, which relieved the symptoms dramatically.

**Lesson:**

The neurovascular cross-compression of the vestibulocochlear nerve by the contralateral vertebral artery tortuosity can cause VP. Periodic paroxysms of right-beating nystagmus accompanying the left-side tinnitus during vertigo attacks in our patient can be explained by secondary central hyperactivity in both vestibular and cochlear nuclei following long-standing neurovascular cross-compression.

## Abstract 2

Supplemental Digital Content is available in the text

## 1 Introduction

Vestibular paroxysmia (VP) is characterized by spontaneous, recurrent, short, paroxysmal attacks of vertigo with or without tinnitus.^[1]^ A neurovascular cross-compression (NVCC) of the vestibulocochlear nerve has been suggested as the underlying cause of VP.^[1]^ The diagnosis of VP is mainly based on the patient history including at least 10 attacks of spontaneous spinning or non-spinning vertigo, duration less than 1 minute, stereotyped phenomenology in a particular patient, response to a treatment with carbamazepine/oxcarbazepine, and not better accounted for by another diagnosis.^[1]^ In this study, we report a patient with VP, which is caused by the contralateral tortuous vertebral artery, having periodic vertigo with paroxysmal nystagmus accompanied by clicking tinnitus in the left side.

## 2 Case presentation

**Figure 1:**
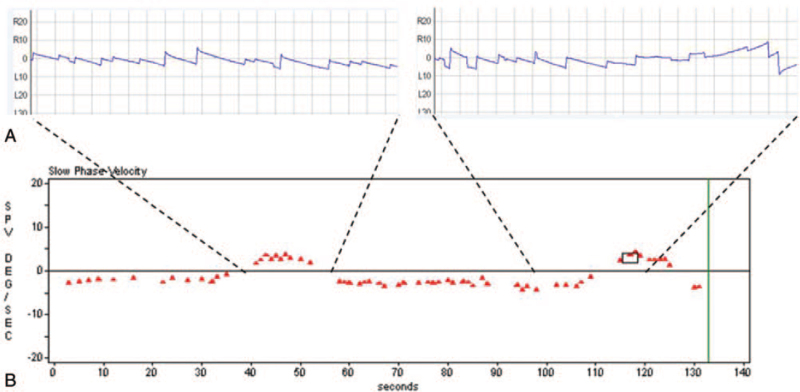
(A) Video-nystagmography demonstrates background right-beating spontaneous nystagmus which is periodically reversed to left-beating nystagmus. (B) The plotting of slow-phase velocity of nystagmus shows that a background right-beating nystagmus is reversed every 55 seconds to left-beating nystagmus which lasts about 17 seconds. Negative value indicates that slow component of nystagmus directs toward the left side. (C) Bithermal caloric tests showed a canal paresis of 83% in the left side. (D) Video head impulse tests revealed significantly decreased vestibulo-ocular reflex gain in the left horizontal semicircular canal.

**Figure 2:**
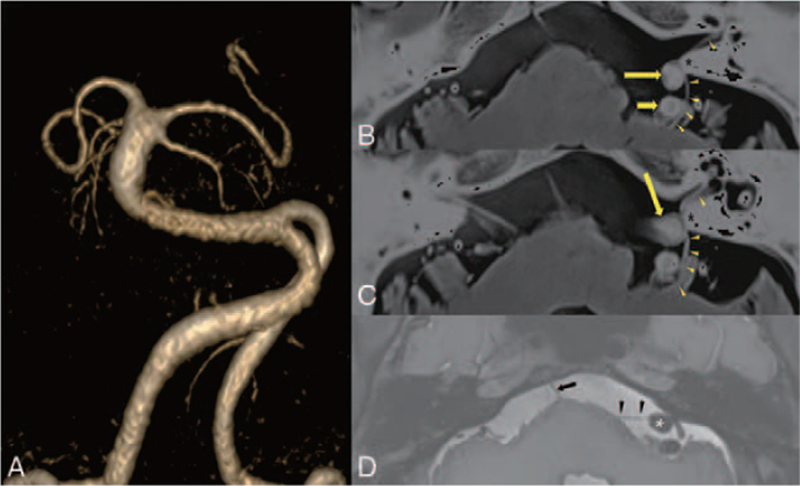
Magnetic resonance imaging (MRI) and angiography (MRA). 3D time-of-flight (TOF) MRA shows laterally displaced course of the distal segments of the intracranial vertebral arteries and mid to proximal segment basilar artery (A). Fusion image of proton density MR cisternography and TOF-MRA shows distal cisternal segment of the left facial nerve (arrowheads in B) and the left vestibulocochlear nerve (arrowheads in C) are jammed in between the laterally deviated dolichoectatic right vertebral artery (long arrow) and posterior edge of the internal acoustic meatus (asterisks). The proximal cisternal segment of the left facial nerve (arrowheads in B) is stretched by the left vertebral artery (short arrow). Axial proton density MR cisternography (D) demonstrates a laterally deviated course of the left abducens nerve (arrowheads) by the right vertebral artery (asterisk) compared to a normal course of the right abducens nerve (arrow) entering Dorello canal.

A 61-year-old man complained of recurrent paroxysmal attacks of spinning vertigo occurring more than 40 times daily, and accompanying “clicking tinnitus” on the left side. He had undergone microvascular decompression (MVD) to treat the left hemifacial spasm 6 years prior at another hospital, and hemifacial spasm was much relieved after surgery. However, paroxysmal vertigo developed 3 years after MVD, which has been aggravated over the last 4 months. Video-nystagmography demonstrated a persistent right-beating nystagmus which was reversed every 55 seconds, to left-beating nystagmus for about 17 seconds, accompanying vertigo (Fig. 1A and B; Video S1, Supplemental Digital Content, http://links.lww.com/MD/G478 [Supplemental video 1. Video-Frenzel examination shows a background right-beating nystagmus which was interposed by a left-beating nystagmus every 55 seconds. The left-beating nystagmus lasts for about 17 seconds]). Hyperventilation or positional maneuver did not affect the pattern of spontaneous and paroxysmal nystagmus. The patient also complained of diplopia during leftward gaze, which had developed 20 years prior, and ocular mobility examination revealed limited abduction of his left eye. Other neurologic examinations were unremarkable. Pure tone audiometry and auditory brainstem response were normal. Bithermal caloric tests showed a canal paresis of 83% in the left side, and video head impulse tests revealed significantly decreased vestibulo-ocular reflex gain in the left horizontal semicircular canal. Interaural amplitude difference ratio of cervical vestibular-evoked myogenic potential was within normal range. Brain magnetic resonance angiography showed markedly tortuous bilateral vertebral artery, and the right vertebral artery crosses the midline, dislocating the left vertebral artery laterally (Fig. 2A). Fusion image of proton density MR cisternography and 3D time-of-flight-magnetic resonance angiography demonstrated distal cisternal segment of the left facial nerve and left vestibulocochlear nerve are jammed in between laterally deviated dolichoectatic right vertebral artery and the posterior edge of the internal acoustic meatus (Fig. 2B and C). The left abducens nerve was laterally deviated by the right vertebral artery (Fig. 2D). Administration of 300 mg oxcarbazepine daily significantly resolved paroxysmal vertigo and tinnitus. The patient reported that his tinnitus symptom disappeared and brief repeated attacks of vertigo subsided from the day after taking oxcarbazepine. Video-nystagmography, which was performed at 2 weeks after the beginning of medication, demonstrated a weak persistent right-beating nystagmus without paroxysmal reversing of nystagmus direction. Any worsening of symptoms was not reported during 7 months of follow-up.

## 3 Discussion

The clinical history of our patient meets the diagnostic criteria for VP.^[1]^ Although the MVD 6 years prior was successful for the left hemifacial spasm, the symptoms of VP newly developed 3 years after MVD. A chronic left peripheral vestibulopathy was evident from background right-beating nystagmus and results of a caloric test and video head impulse test, resulting from chronic vascular compression of the vestibulocochlear nerve. Depending on the duration and locations of NVCC, variable patterns of paroxysmal ipsilesional nystagmus can develop in patients with VP.^[2–6]^ Paroxysms of left-beating nystagmus during the spells of VP in our patient can be explained by secondary central hyperactivity in both vestibular nucleus or direct pulsatile compression with ephaptic discharges in the peripheral vestibular nerve. Grigoryan et al^[7]^ reported a case with hemifacial spasm which was resulted from compression of the facial nerve by the contralateral tortuous vertebral artery. Zang et al^[8]^ reported a case of simultaneous abducens and vestibulocochlear nerve symptoms related to vertebrobasilar dolichoectasia. Considering the limited space within the posterior fossa, the successive occurrence, although MVD had been performed 3 years prior to the onset of VP, of abducens nerve dysfunction, hemifacial spasm and VP is closely related to NVCC by the contralateral tortuous vertebral artery. The present study is, to the best of our knowledge, the first report demonstrating the paroxysmal direction-changing nystagmus in VP caused by the contralateral tortuous vertebral artery. Although randomized prospective placebo-controlled studies on the efficacy of treatments in VP are not available so far, observational studies have demonstrated that a low dose of carbamazepine or oxcarbazepine is effective in the improvement of frequency, intensity, and duration of vertigo attacks.^[1]^

## 4 Conclusion

The NVCC of the vestibulocochlear nerve by the contralateral vertebral artery tortuosity can cause VP. Periodic paroxysms of right-beating nystagmus accompanying the left-side tinnitus during vertigo attacks in our patient can be explained by secondary central hyperactivity in both vestibular and cochlear nuclei following long-standing NVCC.

## Author contributions

**Conceptualization:** Jin Woo Choi, Chang-Hee Kim.

**Data curation:** Jin Woo Choi, Chang-Hee Kim.

**Investigation:** Jin Woo Choi, Chang-Hee Kim.

**Supervision:** Jin Woo Choi, Chang-Hee Kim.

**Writing – original draft:** Jin Woo Choi, Chang-Hee Kim.

**Writing – review & editing:** Chang-Hee Kim.
